# Prevalence, Pattern and Treatment of Traumatic Bone Loss Following Long Bone Open Fractures at Kilimanjaro Christian Medical Centre

**DOI:** 10.24248/eahrj.v8i3.813

**Published:** 2025-01-30

**Authors:** Tangai E Mwanga, Lele M Fabrice, Peter M Magembe, Rogers Temu, Honest Massawe, Octavian Shirima, Faiton N Mandari, Reginald R Shoo

**Affiliations:** a Orthopedics and Traumatology, Kilimanjaro Christian Medical Centre; b Kilimanjaro Christian Medical University College; c General Surgery, Kilimanjaro Christian Medical Centre.

## Abstract

**Background:**

Bone loss resulting from open long bone fractures is a significant concern in low-income countries. This study aims to assess the prevalence, pattern, and treatment of traumatic bone loss in northeastern Tanzania.

**Methods:**

A hospital-based cross-sectional analytical study was conducted at the Orthopedics and Trauma Department of Kilimanjaro Christian Medical Centre (KCMC) from August 2020 to February 2023. The sample size comprised 365 participants with 394 open injuries. Data collection involved a structured electronic questionnaire, and statistical analysis was performed using SPSS version 25.

**Results:**

The study found that 14.7% of the patients treated at KCMC had traumatic bone loss following open long bone fractures. The majority of these patients were young males from rural areas involved in road traffic crashes. Comminuted fractures, especially in the tibia and fibula, were the most common fracture patterns associated with bone loss. Surgical debridement and external fixators were the primary treatment approach for patients with bone loss.

**Conclusion:**

Traumatic bone loss following open long bone fractures is a significant issue in northeastern Tanzania. This study highlights the association between injury pattern, as per Gustilo classification, and the severity of the injury, consequently influencing the treatment plan and the potential for limb salvage. The factors significantly associated with bone loss were the Gustillo IIIB/IIIC classification of open fractures, fractures in the tibia and fibula, fibula fractures, and residence in an urban area.

## BACKGROUND

Bone loss is defined as bone mission or devascularization, periosteal stripping, and bone defects ≥2.5cm.^1^ According to Keating and Robinson, the prevalence of bone loss worldwide ranges from 17% to 40%, with higher rates in developing countries.^2.3^ Kironde et al. found that in East Africa, traumatic bone loss following open long bone fractures is 26.5%, and the leading causes are gunshot injuries, motorcycle accidents, and falls from height.^4^ Commercial motorcycle drivers and traders, with an average age of 30.8 ± 11, are most affected by traumatic bone defects.^5^

Bone loss can have a significant impact on patients’ lives and result in prolonged hospitalisation and financial constraints, leading to inadequate treatment and disability. Therefore, Kironde et al. emphasise the need for increased attention and effort in infrastructure development and advanced specialized management of long traumatic bone open fractures with bone loss in low-income countries. This would aim to reduce hospital stays and improve outcomes, ultimately reducing disability.^1^

The World Health Organization (WHO) has identified road traffic crashes as the leading cause of orthopedic injuries and disability in low-income countries, emphasizing its global public health concern.^1^ Open fractures and bone loss in upper extremities are typically caused by high-energy trauma, gunshot injuries, blunt trauma, or nonunion with infection^6^ Kironde et al. further state that open fractures are common in developing countries due to an increasing number of motor traffic crashes, communal clashes, and civilian gunshot injuries, along with an increased number of unqualified motorcycle drivers.^1^

Treatment for significant bone loss is challenging and often involves complex surgeries with uncertain outcomes.^1^ Keating and Robinson state that open fractures, which account for 0.4% of all fractures, are commonly seen in developing countries due to high-energy injuries from road traffic crashes and motorcycle accidents.^2^ Open fractures may also be complicated by bone defects and bone loss, which can result directly from acute trauma such as road traffic crashes, gunshot injuries, and falls from heights.^4^

Advancements in surgical techniques and the use of antibiotics have brought about changes in the management of open fractures.^7^ The decision to salvage an injured limb depends on the extent of damage to muscle, bone, and joints as well as the potential for neurological and vascular recovery.^8^ Conventional autologous bone grafts are widely used to treat bone defects due to various contributing factors.^9^ The management of bone loss involves debridement, wound closure, fracture stabilization, antibiotics, and tetanus prophylaxis, with the prompt administration of antibiotics to reduce the risk of infection. ^10^

The prevalence of bone loss globally ranges from 17% to 40%, with a specific rate of 26.5% for traumatic bone loss due to open long bone fractures in East Africa.^2,3,11^ There is limited understanding of the prevalence, patterns, and treatments of this condition in northeastern Tanzania, where many trauma patients require management for bone loss. This study aims to address these gaps by highlighting the public health significance, as traumatic bone loss can lead to long-term disability and economic strain. It aims to fill a research void since most existing literature pertains to high-income countries, which may not be relevant to regional healthcare contexts. Furthermore, the study seeks to optimize treatment strategies by analyzing fracture patterns and management methods in local settings. It also focuses on building the capacity of healthcare professionals in orthopedic trauma care, which can improve patient outcomes. Findings from this research may have broader implications for other resource-limited settings facing similar challenges. Overall, the comprehensive evaluation of traumatic bone loss in northeastern Tanzania is crucial for enhancing orthopedic care, developing treatment protocols, and ultimately improving patient health outcomes in low-income settings.

## METHODS

### Study design and Study site

This was a hospital-based Cross-sectional analytical study conducted at the Orthopedics and Trauma Department - Kilimanjaro Christian Medical Centre (KCMC), in northern Tanzania, from August 2020 to February 2023. The facility also serves as a teaching hospital for the Kilimanjaro Christian Medical University College (KCMUCo).

### Sample Size and Sampling Technique

The sample size was estimated by the Kish Leslie formula for cross-sectional studies.^12^ Thus, considering the proportion of traumatic bone loss following open long bone fractures of 26.5% according to Kironde study.^1^ Assuming a confidence level of 95% and a margin of error of 5%, the calculated sample size was 299.

### Study Participants

All patients with open long bone fractures were attended from February 2020 to August 2023.

### Data Collection and Analysis

Data was collected using a well-structured electronic questionnaire The collected information included demographic profile, mode of injury, clinical presentation, site of the fracture, and treatment modalities.

Data were coded and entered into the computer using the SPSS program version 25. Mean and standard deviation (SD) were used to summarize the numerical data. In contrast, frequency and proportions will summarise categorical variables using tables and figures. Relationships between variables were tested using a chi-square test. The odd ratio at a 95% confidence interval was used to test the likelihood of the happening of an event.

### Ethical Consideration

Ethical approval to conduct the study was obtained from the Research and Ethics Committee of KCMUCo, Tumaini University Makumira (Reference Number PG124/2022). The study observed the confidentiality and privacy of the subjects.

## RESULTS

This study involved a total of 365 participants with 394 open injuries. The average age of the participants was 34.9 years with a standard deviation of 15.1. The majority of the participants (48.5%) fell within the age range of 18–34 years. Among the participants, 83.3% were males, 77.4% lived in rural areas, 38.6% had secondary education, 27.9% were peasants, 64.5% were married, 83.5% were non-smokers, 67.5% consumed alcohol, and 87.6% were involved in road traffic crashes (Table 1).

**Table 1:** Social Demographic Characteristics of the Study Participants (n=394)

Characteristics	n (%)
Age (years) (mean (SD)	34.9 (15.1)
Age (years)	
< 18	27 (6.9)
18–34	191 (48.5)
35–44	73 (18.5)
> 44	103 (26.1)
Sex	
Male	328 (83.3)
Female	66 (16.7)
Residence	
Rural	305 (77.4)
Urban	89 (22.6)
Education level	
None	12 (3.1)
Primary	151 (38.3)
Secondary	152 (38.6)
Tertiary	79 (20.0)
Occupation	
Students	40 (10.2)
Drivers	90 (22.8)
Peasant	110 (27.9)
Business	33 (8.4)
Entrepreneurship	101 (25.6)
Government employee	15 (3.8)
Child	5 (1.3)
Marital status	
Not married	140 (35.5)
Married	254 (64.5)
Smoking	
No	329 (83.5)
Yes	65 (16.5)
Alcohol use	
No	128 (32.5)
Yes	266 (67.5)
Mechanism of injury	
RTC	345 (87.6)
Fall	14 (3.5)
Assault	2 (0.5)
Others	33 (8.4)

Among patients treated at KCMC, the burden of traumatic bone loss following open long bone fractures was found to be 14.7%. The median length of bone loss was 3.5 centimeters, with a range of 3.0 to 7.0 centimeters. Among those patients who experienced bone loss, 60.3% were aged 18 to 34 years, 86.2% were males, 89.7% resided in rural areas, 39.7% had primary education, 31.0% were drivers, 63.8% were married, 82.8% did not smoke, 79.3% consumed alcohol, and 91.4% were involved in road traffic crashes. (Table 2)

**Table 2:** Characteristics of the Study Participants by the Burden of Bone Loss (n=394)

Characteristics	Bone loss		Total n (%)	*P Value*
	No n (%) 336 (85.3)	Yes n (%) 58 (14.7)		
	336 (85.3)	58 (14.7)		
Age (years)				
< 18	27 (8.0)	0 (0.0)	27 (6.9)	
18–34	156 (46.4)	35 (60.3)	191 (48.5)	
35–44	65 (19.4)	8 (13.8)	73 (18.5)	
> 44	88 (26.2)	15 (25.9)	103 (26.1)	.059
Sex				
Male	278 (82.7)	50 (86.2)	328 (83.3)	
Female	58 (17.3)	8 (13.8)	66 (16.7)	.514
Residence				
Rural	253 (75.3)	52 (89.7)	305 (77.4)	
Urban	83 (24.7)	6 (10.3)	89 (22.6)	.016
Education level				
None	12 (3.6)	0 (0.0)	12 (3.1)	
Primary	128 (39.1)	23 (39.7)	151 (38.3)	
Secondary	132 (39.3)	20 (34.5)	152 (38.6)	
Tertiary	64 (19.0)	15 (25.9)	79 (20.0)	.315
Occupation				
Students	37 (11.0)	3 (5.2)	40 (10.2)	
Drivers	72 (21.4)	18 (31.0)	90 (22.8)	
Peasant	98 (29.2)	12 (20.7)	110 (27.9)	
Business	30 (8.9)	3 (5.2)	33 (8.4)	
Entrepreneurship	84 (25.0)	17 (29.3)	101 (25.6)	
Government employee	10 (2.9)	5 (8.6)	15 (3.8)	
Child	5 (1.5)	0 (0.0)	5 (1.3)	.085
Marital status				
Not married	119 (35.4)	21 (36.2)	140 (35.5)	
Married	217 (64.6)	37 (63.8)	254 (64.5)	.908
Smoking				
No	281 (83.6)	48 (82.8)	329 (83.5)	
Yes	55 (16.4)	10 (17.2)	65 (16.5)	.869
Alcohol use				
No	116 (34.5)	12 (20.7)	128 (32.5)	
Yes	220 (65.5)	46 (79.3)	266 (67.5)	.038
Mechanism of injury				
RTC	292 (86.9)	53 (91.4)	345 (87.6)	
Fall	14 (4.2)	0 (0.0)	14 (3.5)	
Assault	1 (0.3)	1 (1.7)	2 (0.5)	
Others	29 (8.6)	4 (6.9)	33 (8.4)	.196

Among patients at KCMC with open long bone fractures and traumatic bone loss, the majority (93.1%) had comminuted fractures. In terms of specific bones affected, 58.6% of patients had injuries on the Tibia and fibula. Furthermore, 77.6% had Castillo III C injuries, and 63.8% had injuries on the distal portion of the body segment. (Table 3)

**Table 3:** The Patterns of Open Long Bone Fractures with Traumatic Bone Loss among Patients Treated at KCMC (n=394)

Characteristics	Bone loss		Total n (%)	*P Value*
	No n (%)	Yes n (%)		
	336 (85.3)	58 (14.7)		
Types of fractures				
Transverse	84 (25.0)	0 (0.0)	84 (21.3)	
Comminuted	174 (51.8)	54 (93.1)	228 (57.9)	
Oblique	52 (15.5)	2 (3.4)	54 (13.7)	
Spiral	13 (3.9)	0 (0.0)	13 (3.3)	
Segmental	13 (3.9)	2 (3.5)	15 (3.8)	<.001
Fractured bone				
Femur	25 (7.4)	12 (20.7)	37 (9.4)	
Tibia/fibula	246 (73.2)	34 (58.6)	280 (71.1)	
Tibia	25 (7.4)	5 (8.6)	30 (7.6)	
Fibula	18 (5.4)	1 (1.7)	19 (4.8)	
Humerus	7 (2.1)	3 (5.2)	10 (2.5)	
Radius/Ulnar	15 (4.5)	3 (5.2)	18 (4.6)	.015
Gustillo's classification of open fractures				
I	16 (1.8)	0 (0.0)	6 (1.5)	
II	155 (46.1)	0 (0.0)	155 (39.3)	
IIIA	134 (39.9)	2 (3.5)	136 (34.5)	
IIIB	32 (9.5)	11 (18.9)	43 (10.9)	
IIIC	9 (2.7)	45 (77.6)	54 (13.7)	<.001
Fracture location				
Proximal	71 (21.2)	15 (25.9)	86 (21.9)	
Middle	57 (17.0)	6 (10.3)	63 (16.0)	
Distal	207 (61.8)	37 (63.8)	244 (62.1)	0.387

The study revealed that the most common treatment approach for participants was surgical debridement (SD) and external fixator (EXFX), utilized by 47.5% of participants. The second most prevalent treatment was SD and back slab, chosen by 36.6% of participants. Other treatment methods, such as SD with K-wire, nail, bone cement, and EXFX, SD with rush rod, K-wire, back slab, EXFX, and traction, or amputation, were less commonly employed, ranging from 2.3% to 5.3%. In cases where bone loss was present, 58.6% of participants opted for SD and EXFX treatment, while 15.5% chose SD with back slab and amputation. The least frequently used treatment modality, accounting for only 1.7% of cases, involved a combination of SD, rush rod, K-wire, and back slab. (Table 4)

**Table 4:** Treatment Options by Bone Loss (n=394)

	Bone loss		Total n (%)	*P Value*
	No n (%)	Yes n (%)		
	336 (85.3)	58 (14.7)		
Treatment options				
SD and back slub	135 (40.2)	9 (15.5)	144 (36.6)	
SD and EXFX	153 (45.5)	34 (58.6)	187 (47.5)	
SD, K-wire, nail, bone cement and EXFX	18 (5.4)	3 (5.2)	21 (5.3)	
SD, rush rod, k-wire and backslub traction and hip Spika	11 (3.3)	1 (1.7)	12 (3.1)	
	1 (0.3)	0 (0.0)	1 (0.3)	
Amputation	0 (0.0)	9 (15.5)	9 (2.3)	
SD without EXFX	4 (1.2)	0 (0.0)	4 (1.0)	
K-wire and EXFX	3 (0.9)	0 (0.0)	3 (0.8)	
SD, EXFX and traction	10 (2.9)	2 (3.5)	12 (3.1)	
SD, K-wire and backslub	1 (0.3)	0 (0.0)	1 (0.3)	<.001

SD: surgical debridement, EXFX: External Fixator

The study found several significant factors associated with bone loss. Gustillo IIIB/IIIC fractures were positively associated with bone loss, with an adjusted odds ratio (AOR) of 1.81 and a 95% confidence interval (CI) of 1.06–7.70 (*P* <.001). On the other hand, tibia and fibula fractures were negatively associated with bone loss, with an AOR of 0.24 and a 95% CI of 0.11–0.55 (*P* =.001). Similarly, fibula fractures alone were negatively associated with bone loss, with an AOR of 0.057 and a 95% CI of 0.01–0.79 (*P* =.001). Urban residence was also negatively associated with bone loss, with an AOR of 0.30 and a 95% CI of 0.13–0.74 (*P* =.009). Other factors examined in the study did not show significant associations with bone loss. (Table 5, Table 6)

## DISCUSSION

This study aimed to evaluate the prevalence, pattern, and treatment of traumatic bone loss following open long bone fractures treated at tertiary care hospitals in northeastern Tanzania, focusing on the Orthopedic and Trauma Department. A total of 365 study participants with 394 open injuries were included, with a mean (SD) age of 34.9 (15.1) years. Similar distribution of age and injury characteristics was observed in studies conducted in Scotland. ^1,2^

Regarding the participants’ backgrounds, the majority of those with open fractures hailed from rural settings. However, it was observed that most participants affected by bone loss resided in urban areas. Moreover, patients with tertiary education displayed lower incidence of traumatic bone loss following open long bone fractures compared to those with primary and secondary education, although those with primary education demonstrated a relatively higher incidence overall.

The burden of traumatic bone loss following open long bone fractures among patients treated at the study hospital was found to be 14.7% (58 cases). Similar prevalence rates were reported in studies conducted in Scotland and Uganda.^1,2^

In terms of fracture patterns, the majority of individuals with bone loss (93.1%) had comminuted fractures, with 58.6% of these occurring in the tibia and fibula. Additionally, 77.6% presented with Castillo III C injuries, and 63.8% sustained injuries on the distal portion of the bone segment. This pattern aligns with findings reported by Dugan et al.^13^

This study highlights the association between injury pattern, as per Gustilo classification, and the severity of the injury, consequently influencing the treatment plan and the potential for limb salvage. The segmental fracture pattern was found to be particularly associated with bone loss compared to other open long bone fracture patterns.

Furthermore, individuals aged 18–34 years and males exhibited a higher prevalence of traumatic bone loss following open long bone fractures compared to females.

In terms of treatment, a significant proportion of those with bone loss (58.6%) at the study hospital were treated with surgical debridement (SD) and external fixator, followed by 15.5% treated with surgical debridement (SD) and back slab, and a minor proportion (1.7%) treated with surgical debridement (SD), rush rod, k-wire, and back slab. These treatment approaches align with studies conducted in the UK and China.^8,14^

The primary treatment approach for traumatic bone loss following open long bone fractures in this study involved surgical debridement with an external fixator, followed by the application of a cast in regular clinics after two to four weeks. Additionally, some individuals received treatment involving surgical debridement and membrane-induced techniques, while a few required amputations due to significant bone loss.

Strength: This study provides valuable insights into the burden, pattern, and treatment of traumatic bone loss following open long bone fractures in a previously unstudied region, KCMC.

Limitations: The study is limited by the by the fact that small fragments of bone loss in comminuted fractures may have been missed during intraoperative assessment, potentially leading to an underestimation of the extent of bone loss. The study also did not assess the amount of bone defect pre-surgery and post-debridement, making it difficult to determine the relative contribution of surgical treatment versus the initial bone loss.

## CONCLUSION

In conclusion, traumatic bone loss following open long bone fractures presents a significant public health issue in northeastern Tanzania, impacting 14.7% of patients treated at KCMC. The majority of those affected are young, male, rural residents, particularly involved in road traffic accidents. The most prevalent patterns of bone loss involve comminuted fractures of the tibia and fibula, affecting the lower extremities, with severity often classified as Gustillo IIIB/IIIC for open fractures. The primary treatment method utilized is surgical debridement in conjunction with external fixation. Notably, factors such as the severity of fractures, specific anatomical locations, and urban residency are significantly associated with traumatic bone loss.

These findings underscore the urgent need to address this issue through effective management and treatment strategies to enhance recovery outcomes for affected patients. The insights gained from this study are essential for healthcare practitioners and policymakers in developing targeted interventions that can improve patient care and outcomes in similar resource-limited settings. By focusing on the prevalent demographic and clinical factors involved, healthcare systems can better allocate resources and implement effective treatment protocols for traumatic bone loss.
